# Extracorporeal cardiopulmonary resuscitation for severe chloroquine intoxication in a child – a case report

**DOI:** 10.1186/s13049-021-00850-0

**Published:** 2021-03-15

**Authors:** Thomas Ferry, Vivianne Amiet, Julia Natterer, Marie-Hélène Perez, Raymond Pfister, Sébastien Colombier, David Longchamp

**Affiliations:** 1 Paediatric Intensive Care, Lausanne University Hospital, University of Lausanne, Rue du Bugnon 46, CH-1011 Lausanne, Switzerland; 2 Department of Cardiovascular Surgery, Lausanne University Hospital, University of Lausanne, Lausanne, Switzerland

**Keywords:** Pediatrics, Extracorporeal Life support, Resuscitation, Emergency medicine, Intensive Care, Arrhythmia

## Abstract

**Background:**

Chloroquine use has increased worldwide recently in the setting of experimental treatment for the novel coronavirus disease (Covid-19). Nevertheless, in case of chloroquine intoxication, it can be life threatening, with cardiac arrest, due to its cardiac toxicity.

**Case presentation:**

This case study reports on a 14-years-old girl who presented in cardiac arrest after an uncommon suicide attempt by ingesting 3 g of chloroquine. After 66 min of cardio-pulmonary resuscitation (CPR), extracorporeal cardiopulmonary resuscitation (ECPR) was initiated, allowing cardiac function to recover.

**Conclusions:**

Chloroquine intoxication is a rare but serious condition due to its cardiac toxicity. Use of ECPR in this case of transient toxicity allowed a favorable evolution with little neurological impairment.

## Background

Chloroquine is a worldwide recognized treatment for varied conditions such as malaria, rheumatoid arthritis and lupus erythematosus. Its use has recently been significantly expanded as an experimental treatment for COVID-19. Overdose however, can lead to life threatening complications [1], and with the lack of a specific antidote, treatment remains supportive, extracorporeal membrane oxygenation (ECMO) being the last resort. We report the case of a teenager who suffered cardiac arrest due to chloroquine, and who survived to ECMO decannulation. To our knowledge, this is the first published pediatric case of chloroquine intoxication requiring extracorporeal cardiopulmonary resuscitation (ECPR).

## Case presentation

A 14-years-old girl, with a weight of 68 kg and a history of suicidal thoughts presented in cardiopulmonary arrest following a suicide attempt with ingestion of 3 gram of chloroquine without history of any other substance ingestion. Upon the arrival of the ambulance, she was unconscious with a Glasgow coma scale score (GCS) of 6 (1–1–4), bradypneic and with a trismus. The electrocardiogram (ECG) showed ST-segment changes, with pulseless ventricular tachycardia immediately following. Cardio-pulmonary resuscitation was initiated but despite chest compressions, defibrillation and adrenaline, there was no return to spontaneous circulation. Twelve minutes later, upon arrival at the hospital, she had a pulseless idioventricular rhythm and cardiopulmonary resuscitation was continued. The patient was intubated and external massage was taken over by a Lucas® chest compression system (Stryker Medical, Portage, MI49002 US).

Blood gas revealed severe hypokalemia (potassium 1.8 mmol/l) and a severe mixed acidosis (pH 6.97, pCO2 95 mmHg, glucose 14.5 mmol/l, lactate 7.4 mmol/l, base excess − 9.7 mmol/l, bicarbonate 12.7 mmol/l). Resuscitation was continued with three additional doses of adrenaline followed by continuous adrenaline infusion as well as correction of hypokalemia, administration of an amiodarone bolus, a bicarbonate bolus, fluid resuscitation and intravenous lipid emulsion infusion. Despite these measures, resuscitation was unsuccessful.

Peripheral right femoral veno-arterial extracorporeal membrane oxygenation (ECMO)ʼ was therefore initiated after 66 minutes of resuscitation to provide full cardiac support. During transfer to our pediatric intensive care unit (PICU), she was sedated and paralyzed, ventilated, hemodynamically stable on ECMO with a blood flow of 1.7 L/min/m^2^ and an adrenaline infusion of 0.1mcg/kg/min, a temperature of 34.9 °C. Cerebral computed tomography scan performed on admission was normal. On arrival in PICU, cardiac ultrasound on ECMO showed left ventricular dysfunction with an estimated ejection fraction of 35 %, no left or right ventricular dilatation and without mitral valve regurgitation. Plasma hydroxychloroquine level taken a few hours after PICU admission (equal to ten hours post ingestion) was 0.06mcmol/L. Urinary toxic screening was positive for THC/cannabinoid, benzodiazepine and opioid (benzodiazepine and opioid being administered during initial medical care) and negative for acetaminophen, amphetamine/metamphetamine, barbiturate, cocaine, methadone, phencyclidine, tricyclic antidepressor.

ECMO blood flow was increased to 2L/min/m2 and adrenaline infusion weaned shortly after her admission, and heparin infusion started with ACT target range of 180–220. Targeted temperature management in the range of 34–35 °C was done for 48 h. ECMO course was uneventful. Cardiac ultrasound 46 hours post event showed recovery with systolic ejection fraction of 52 % and mild right diastolic dysfunction, allowing weaning from ECMO. A few hours later, a poor perfusion of the right lower limb was observed with a vascular doppler ultrasound revealing a significant reduction of arterial flow of the right common femoral artery. Immediate wound exploration revealed right common femoral artery stenosis without thrombosis at the site of the cannula insertion. Consequently, an arterial vascular surgical reconstruction was done with a venous patch. She developed a compartment syndrome of the right leg in the hours following, requiring fasciotomy.

When sedation was discontinued on day 4, the patient showed minimal interaction and no intentional movement. Striatal lesions were described on cerebral magnetic resonance imaging (MRI). Electroencephalogram (EEG) on day 6 revealed moderate reactive encephalopathy. On day 7, significant neurological improvement was observed, and the patient was extubated. Neurological exam revealed full consciousness, good spatiotemporal orientation, some memory deficit, and no focal neurologic deficit except hypoesthesia L5-S1 of the right foot and a right elevator muscle deficit secondary to right leg compartment syndrome. She was discharged from PICU at day 11 and then transferred from our tertiary center to her local rehabilitation hospital to continue intensive neuro-muscular physiotherapy.

## Discussion

Chloroquine intoxication is a rare condition, associated with severe cardiotoxicity due to its quinidine-like properties. It is a strong membrane stabilizer acting like a class Ia antiarrhythmic agent (action on voltage-dependent sodium channel). Symptoms appear from two to three hours post ingestion and usually resolve within 24 hours, despite a long half-life (14 days). Cardiac toxicity is the result of the rapid rise in chloroquine plasma level during the first two hours, but it can extend to the first twenty-four hours. Cardiac toxicity includes negative inotropism, inhibition of spontaneous depolarization, slowing of atrioventricular conduction, increasing of the refractory period, prolongation of the QT segment and QRS interval, Torsades de pointes and multiple ventricular arrhythmias [2]. An ingestion of more than 20 mg/kg is considered a toxic dose with a lethal dose if it’s over 30 mg/kg. More than 4 grams of chloroquine ingested, chloroquine plasma levels > 25 mcmol/L and hypokalemia have been linked to poor prognosis [3], the severity of hypokalemia being related to the severity of the intoxication. Rebound hyperkalemia can be observed after aggressive correction so hypokalemia treatment should be cautious. Chloroquine also affects the respiratory, neurological (irritability, drowsiness, dystonia and seizures) and digestive systems and metabolic acidosis is common.

In our patient, severe intoxication had to be considered, with potentially more than 40 mg/kg of chloroquine ingested. The clinical presentation, similar to the above literature, confirmed the overdose: respiratory depression and neurological symptoms (drowsiness and dystonia), followed by pulseless ventricular tachycardia and cardiovascular collapse, profound metabolic acidosis and severe hypokalemia. Surprisingly, hydroxychloroquine plasma level was much lower (0,06mcmol/L) than the toxic levels found in literature (usually around 10–30 mcmol/L). We hypothesize that three reasons may explain this result. The first is possible adsorption of chloroquine by the ECMO system (tubing and oxygenator) or binding by of the intravenous lipid emulsion treatment. The second is the hemodilution by both the ECMO circuit and the fluid administration during CPR and on ECMO support. The last is the quality of the sample itself, possibly altered by dilution or sampling procedure error. In our patient, the chloroquine plasma level was not clinically relevant as management was driven by the patient’s condition.

Overdose cases remain rare, so there are no strong recommendations for management. However, from the existing literature, specific treatment combines assisted ventilation and administration of diazepam, adrenaline and intravenous lipid emulsion [2, 4]. Diazepam administration is controversial. It is part of the supportive treatment: used for sedation, in case of seizures and for its presumed antiarrhythmic properties [4]. However, there is no evidence that this treatment alone, as a potential antidote, significantly improves the outcome of moderately intoxicated patients. Adrenaline counteracts vasodilation and myocardial depression, playing a key role in resuscitation of the severely intoxicated patients [2]. Our patient received both treatments (adrenaline and diazepam), before stabilization on ECMO.

Intravenous lipid emulsion has been used in systemic anesthetic toxicity and in poisoning with other lipophilic drugs. As chloroquine is highly lipophilic, the early use of intravenous lipid emulsion in chloroquine intoxication could possibly reduce its plasma peak level of chloroquine and therefore reduce its toxicity. Our patient received a bolus followed by a continuous, but it was rapidly stopped when on ECMO support; indeed, ECMO is a relative contraindication due to a potential obstructive effect on oxygen filter, fat emulsion agglutination and increased blood clot formation in the circuit [5].

When given early enough after ingestion, implying the time of ingestion is known, activated charcoal could prevent absorption of any chloroquine remaining in the stomach. The use of intravenous bicarbonate is mentioned in case of widening of QRS complex. Hemodialysis and hemoperfusion on the other hand are not effective due to the high volume of distribution of chloroquine, therefore these modalities were not considered in our patient [6]

As chloroquine intoxication is a reversible phenomenon, mainly causing symptoms of direct cardiotoxicity, rapid efficient advanced cardiac life support (ACLS) is key to its management, including ECPR. ECMO is described as an option for selected poisoned patients, as it provides organ support during the acute phase of intoxication [7]. Available data show that the use of ECPR offers the possibility of survival with good neurologic recovery in adult out-of-hospital cardiac arrest (OHCA) of varying causes [8]. A shockable rhythm, female gender, short no flow time or witnessed cardiac arrest, short low-flow time and good quality CPR seem to play a positive role on outcome despite ongoing discrepancy about these prognostic factors in the literature [8]. Furthermore, the outcome of ECPR is improved when provided by experienced and trained centers. ECPR is currently provided on a case-by-case basis, where it can be quickly implemented and in patients for whom the etiology of the cardiac arrest is potentially reversible within a limited period of mechanical cardiorespiratory support [7]. For the pediatric population, ECPR use is described for in-hospital cardiac arrest (IHCA) [9–14] and mainly related to children with underlying cardiac disease or after cardiac surgery. Pediatric ECPR has a high mortality, with survival to decannulation and to hospital discharge of 58 % and 42 % respectively in the last ELSO registry report [15]. For OHCA and intoxication cases, data on children are, on the contrary, very sparse. Despite high mortality in pediatric ECPR, selected intoxication cases might, in our opinion, benefit from ECPR support because of their reversibility, as illustrated in this case.

In summary, outcome of our patient depended on the patient’s favorable prognostic factors, the quality of initial resuscitation and the experience of all the staff involved in the ECMO support. Despite morbidity linked to sustained muscle weakness of her right lower limb secondary to arterial ischemia, the overall neurological outcome was favorable, considering the severity of the insult and the prolonged resuscitation.

## Conclusions

Chloroquine intoxication can be life threatening, with cardiac arrest, due to cardiotoxicity. Its management is mainly supportive as no antidote is available. This patient fulfilled criteria for optimal use of ECPR, despite the lack of strong evidence for this procedure in intoxication and OHCA in children. Patient criteria and specific protocols regarding use of ECPR are still under study, aiming to improve outcome after pediatric OHCA. Severe reversible intoxication could be one of its indications and should be considered on a case-by-case basis.

## Data Availability

The data used for this case report are part of the personal clinical electronic file and are not pubicly available for confidentiality reasons, but anonymous data are available from the corresponding author on reasonable request.

## Abbreviations

Covid-19: Novel coronavirus disease
CPR: Cardio-pulmonary resuscitation
ECPR: Extracorporeal cardiopulmonary resuscitation
ECMO: Extracorporeal membrane oxygenation
GCS: Glasgow coma scale score
ECG: Electrocardiogram
PICU: Pediatric intensive care unit
MRI: Magnetic resonance imaging
EEG: Electroencephalogram
ACLS: Advanced cardiac life support
OHCA: Out-of-hospital cardiac arrest
IHCA: In-hospital cardiac arrest
